# Evaluation of Clinical SYNTAX Score in Predicting New-Onset Atrial Fibrillation and Long-Term Prognosis in Patients Hospitalized with Non-ST Segment Elevation Myocardial Infarction

**DOI:** 10.3390/jcdd13100488

**Published:** 2026-09-29

**Authors:** Zubeyir Bulat, Mehmet Emin Gokce, Sukru Arslan, Muhammed Heja Gecit, Mehmet Emin Bilgin, Abdullah Omer Ebeoglu, Umit Yasar Sinan, Muhammed Furkan Deniz, Servet Batıt, Veysel Oktay

**Affiliations:** 1 Cardiology Department, Şırnak State Hospital, Nucan Street, No: 34, Bahçelievler Neighborhood, 73000 Şırnak, Turkey; mehmet_emin_gkc@hotmail.com; 2 Institute of Cardiology, Istanbul University-Cerrahpaşa, General Abdurrahman Nafiz Gürman Street, Building No: 24, Cerrahpaşa Neighborhood, Fatih, 34098 Istanbul, Turkey; hejagecit@hotmail.com (M.H.G.); mehmeteminbilgin47@gmail.com (M.E.B.); drumityasar@hotmail.com (U.Y.S.); 3 Bağcılar Training and Research Hospital, Dr. Sadık Ahmet Street, No: 5, Merkez Neighborhood, Bağcılar, 34200 Istanbul, Turkey; ebeoglu995@gmail.com (A.O.E.); drfurkandeniz@gmail.com (M.F.D.); 4 Cardiology Department, Biruni University Faculty of Medicine Hospital, Halkalı Street No: 99, Gültepe Neighborhood, Küçükçekmece, 34295 Istanbul, Turkey; servet.batit@gmail.com

**Keywords:** clinical SYNTAX score, NOAF, atrial fibrillation, NSTEMI-ACS

## Abstract

New-onset atrial fibrillation (NOAF) is a frequent complication among patients with non-ST-elevation myocardial infarction (NSTEMI) and is associated with adverse outcomes, making early identification of predictors essential for risk stratification and management. This retrospective cohort study evaluated the prognostic value of the Clinical SYNTAX score (CSS) compared with the anatomical SYNTAX score (SS), GRACE, and CHA2DS2-VASc scores in predicting in-hospital NOAF and long-term outcomes among 979 NSTEMI patients undergoing coronary angiography. Predictors of NOAF, all-cause mortality, and major adverse cardiovascular events (MACEs: death, myocardial infarction, stroke) were identified using logistic and Cox regression models, with receiver operating characteristic (ROC) analysis used to compare the discriminative power of CSS against SS, GRACE, and CHA2DS2-VASc over a median follow-up of 86 months (IQR 36–100; range 0.1–144). NOAF developed in 80 patients (8.2%), with age, female gender, left atrial diameter, left ventricular diastolic dysfunction, and CSS (*p* = 0.014) identified as independent predictors. CSS’s AUC for predicting NOAF (0.724; 95% CI: 0.673–0.775) was significantly higher than SS’s (0.644; 95% CI: 0.588–0.699; DeLong *p* < 0.001) and comparable to that of the CHA2DS2-VASc and GRACE scores; a CSS threshold ≥ 17.77 predicted NOAF with 75% sensitivity and 65% specificity. In Cox regression analysis, in-hospital NOAF, age, hypertension, reduced ejection fraction (<60%), and CSS independently predicted long-term all-cause mortality, while NOAF, EF < 60%, age, early AF recurrence, hypertension, and CSS independently predicted MACEs; SS was not a significant predictor in either model. In non-overlapping multivariable models, this advantage reflected the contribution of CSS’s clinical components rather than incremental value of the composite score; CSS nonetheless remained an independent predictor of NOAF and long-term outcomes and may serve as a practical single-score tool for risk-guided management.

## 1. Introduction

Despite improvements in early diagnosis and prevention, coronary artery disease remains the leading global cause of death, largely due to its increasing incidence. Acute coronary syndrome typically results from coronary thrombosis triggered by plaque rupture or erosion [1,2]. Atrial fibrillation (AF) is the most common persistent arrhythmia in the adult population, accounting for approximately 33% of hospital admissions due to cardiac arrhythmias. Its prevalence has increased over the years, driven by extended life expectancy and intensified diagnostic efforts for previously undiagnosed AF.

NOAF in ACS patients has declined from approximately 18% in 1990 to 7–12% in recent years, largely due to advancements in thrombolytic, interventional, and pharmacological therapies [3,4,5]. AF is important not only because of its symptoms but also its effects. It increases the risk of stroke five times, doubles the risk of death and cognitive decline, and is linked to worsening heart failure and sudden death [6]. Given these risks, early identification of patients prone to NOAF has gained importance. However, current risk stratification tools specific to NOAF are limited, especially in the context of NSTEMI [7,8]. In such patients, relying solely on the anatomical SYNTAX (Synergy Between Percutaneous Coronary Intervention with Taxus and Cardiac Surgery) score to guide revascularization may be insufficient, as it neglects clinical factors that influence both arrhythmic and long-term outcomes. Comorbidities like hypertension, diabetes, and hyperlipidemia, which can accelerate coronary artery disease progression and its recurrence after revascularization, are essential considerations. Studies have shown that risk scores incorporating clinical parameters, rather than relying solely on anatomical data, provide a more robust prediction of long-term outcomes [9,10,11]. Among these, the ACEF score—which includes age, creatinine, and ejection fraction—has gained popularity as a rapid bedside risk stratification tool. In a multicenter study involving patients with cardiogenic shock, an ACEF score ≥ 2.24 predicted in-hospital mortality with 74% sensitivity and 77% specificity [12]. However, anatomical scoring tools like the original SYNTAX score, while useful in assessing coronary lesion complexity, lack clinical context, which may limit their prognostic accuracy—especially in patients with multiple comorbidities. To overcome these limitations, the SYNTAX II and Clinical SYNTAX score (CSS) systems were developed, integrating detailed anatomical information with clinical risk indices such as ACEF. In patients with STEMI complicated by cardiogenic shock, the SYNTAX II score was shown to be an independent predictor of in-hospital mortality, with patients in the highest tertile exhibiting significantly higher risk [13]. The SYNTAX II and CSS models have since been validated in various clinical settings as more comprehensive and individualized tools for decision-making in coronary artery disease [14].

Inflammation is increasingly recognized as a key pathophysiological mechanism underlying the development of NOAF and its association with major adverse cardiovascular events (MACEs). The uric acid/albumin ratio (UAR), a composite marker reflecting both oxidative stress and nutritional status, has been shown to independently predict AF recurrence after cryoballoon ablation [15] and to outperform traditional biomarkers in forecasting NOAF in patients with ST-elevation myocardial infarction [16]. These findings suggest that atrial remodeling driven by inflammation may help explain the strong prognostic link between NOAF and adverse cardiovascular outcomes. Identifying high-risk patients for NOAF in NSTEMI–ACS could help guide prophylactic anti-arrhythmic treatment and management of modifiable risk factors to reduce long-term mortality. Therefore, assessing predictors of in-hospital NOAF and the impact of the Clinical SYNTAX score, which incorporates clinical parameters, on patient outcomes would be crucial.

## 2. Materials and Methods

### 2.1. Study Design

Our study is a single-center, retrospective cohort study. The study was approved by the Cerrahpaşa Non-Interventional Clinical Research Ethics Committee, as deemed ethically appropriate, under approval number 2023/159, dated 23 November 2023, with reference number E74555795–050.01.04–842099–. The study protocol was prepared in accordance with the Declaration of Helsinki. The study included a review of patient records from the Istanbul University-Cerrahpaşa Cardiology Institute, focusing on those diagnosed with NSTEMI who met the 4th Universal Myocardial Infarction Diagnostic Criteria between January 2011 and December 2019 [17].

### 2.2. Data Acquisition, Exclusion Criteria Implementation, and Follow-Up

#### 2.2.1. Inclusion Criteria

1. Age ≥ 18 years;
2. Admission for NSTEMI fulfilling 4th Universal MI criteria with symptom onset <24 h;
3. Diagnostic coronary angiography ≤48 h after admission;
4. Transthoracic echocardiography and full laboratory panel within 48 h;
5. Complete electronic data for predefined variables.

#### 2.2.2. Exclusion Criteria

1. Documented AF/flutter or long-term anti-arrhythmic use;
2. Prior CABG or valve surgery, or moderate–severe valvular disease;
3. Non-atherosclerotic culprit (spasm, embolism, dissection);
4. Dialysis or creatinine clearance (Cockcroft–Gault) < 15 mL/min;
5. Active infection, systemic inflammatory disease, malignancy, or thyroid dysfunction;
6. Severe hepatic impairment (Child–Pugh C);
7. Hemodynamic instability precluding standard monitoring/telemetry;
8. Missing key clinical, angiographic, or follow-up data.

### 2.3. Patient Enrollment and Flowchart

A total of 1355 patients diagnosed with NSTEMI were identified through the data system of Istanbul University-Cerrahpaşa Cardiology Institute.

- A total of 197 patients were excluded due to prior CABG surgery.
- Twenty-seven patients did not undergo coronary angiography and were excluded.
- A total of 147 patients had a documented history of AF or had undergone prior valve surgery and were excluded.
- Five patients were excluded due to inaccessible data.
- Some of the 147 patients excluded for known AF or prior valve surgery also had angiographically normal coronary arteries, which would independently have warranted exclusion given the potential for diagnostic confusion with Type 2 myocardial infarction; because exclusion categories were not mutually exclusive and were not tagged with multiple reasons at the time of screening, the exact number of patients with normal coronary arteries, and their in-hospital NOAF status, could not be retrospectively determined. Patients with non-obstructive coronary atherosclerosis (visible plaque without any ≥50% stenosis; SYNTAX score = 0, *n* = 104, all managed medically) were retained, as plaque rupture in non-obstructive lesions is a recognized mechanism of Type 1 NSTEMI; none of these 104 patients developed in-hospital NOAF.

Thus, 979 patients were included in the final analysis. The patient selection process is detailed in Figure 1. To enhance external validity and reflect real-world NSTEMI management practices, the cohort intentionally included patients who underwent PCI, CABG, or conservative medical treatment following angiographic evaluation. The inclusion of CABG patients was methodologically justified, as the SYNTAX and Clinical SYNTAX scores—key components of this study—are routinely used to guide revascularization decisions. Their inclusion enabled a comprehensive assessment of these scores across the full spectrum of clinical management strategies. Post-CABG NOAF may arise through partly distinct mechanisms (e.g., cardiopulmonary bypass, atriotomy) compared with PCI- or medically managed patients; a treatment-stratified sensitivity analysis addressing this heterogeneity is reported in Section 3.2 and discussed as a limitation of the pooled NOAF model.

### 2.4. Clinical Data Collection and Scoring Methods

Vital signs, ECG recordings, and laboratory values were collected from the hospital automation system.

- Renal function was expressed as estimated creatinine clearance (CrCl, mL/min) calculated with the Cockcroft–Gault formula.
- Anemia was defined based on WHO criteria (Hb < 13 g/dL in men and <12 g/dL in women).
- GRACE scores were recorded or retrospectively calculated based on available data, using the original GRACE risk model as described by Fox et al. [18]; the GRACE 2.0 model was not used in this study.
- Coronary angiograms were reviewed in the catheterization lab, and SYNTAX scores were calculated via a web-based tool [19] from the baseline diagnostic angiogram prior to percutaneous intervention. Scores were calculated independently by two cardiologists blinded to clinical outcomes, with discrepancies resolved by consensus.
- CSS was computed as the anatomical SYNTAX score multiplied by the modified ACEF score, where modified ACEF = age (years)/left ventricular ejection fraction (%) + 1 point for each 10 mL/min decrease in creatinine clearance below 60 mL/min [20].

### 2.5. Monitoring and Follow-Up

- All patients were continuously monitored during their initial 24 h stay in the coronary intensive care unit (CICU).
- Daily ECGs were obtained after transfer to the ward, with additional ECGs ordered for symptomatic patients.
- Based on progress notes and discharge summaries, patients were categorized according to the occurrence of in-hospital arrhythmic events.
- NOAF was defined as AF or atrial flutter lasting ≥30 s, detected during hospitalization in patients without a prior diagnosis of AF and confirmed by two cardiologists. Patients with prior paroxysmal AF identified only by implantable devices were excluded.
- Atrial flutter was included due to its similar pathophysiology, clinical course, and treatment strategies.

### 2.6. Prognostic Evaluation

The predictive value of the Clinical SYNTAX score in identifying in-hospital NOAF was compared against conventional scoring systems (SYNTAX, GRACE, CHA2DS2-VASc). For long-term prognostic analysis, events such as mortality, recurrent MI, and ischemic stroke, along with their durations, were recorded using a combination of the national health system database and the hospital information system. All-cause mortality was ascertained through the national health system database, ensuring complete nationwide capture regardless of the location of death. Non-fatal events (MI and stroke) were similarly ascertained using the combination of the national health system database and hospital records. Patients who died during the index hospitalization, including within the first 24 h of admission, were included in the follow-up analysis as an event. These outcomes were analyzed in relation to CSS and conventional scoring tools.

### 2.7. Statistical Modeling Strategy

To identify independent predictors of in-hospital NOAF and long-term outcomes, a structured multivariable modeling approach was used. All variables with a *p* value < 0.10 in univariate analyses or established biological plausibility were entered into the multivariable models. Multicollinearity was assessed using the variance inflation factor (VIF), and all included variables had a VIF < 2.

For the prediction of in-hospital NOAF, a forward likelihood-ratio logistic regression model was used. For long-term outcomes (including all-cause mortality and the composite endpoint of death, myocardial infarction, and stroke), Cox proportional-hazards models with stepwise backward elimination were employed. The proportional-hazards assumption was tested using Schoenfeld residuals and found to be met.

Missing data, which accounted for less than 5% of the dataset overall and were confined to variables not entered into the multivariable models (e.g., HbA1c, and the triple antithrombotic therapy field, which had been recorded only for patients with NOAF and is therefore reported in the Results as derived from concurrent aspirin, P2Y12-inhibitor, and oral anticoagulant prescription), were addressed using multiple imputation where relevant. All covariates and outcomes entered into the multivariable regression models (see Results) had complete data (0% missing); the corresponding point estimates and *p* values therefore reflect complete-case analysis rather than pooled multiply imputed estimates.

### 2.8. Statistical Analysis

The statistical analysis of our study was performed using IBM SPSS Statistics version 22.0 (IBM Corp., Armonk, NY, USA). Normality of the data was assessed using the Shapiro–Wilk (W) test. Continuous variables that exhibited normal distribution were analyzed using Student’s *t*-test, while those that did not follow a normal distribution were analyzed using the Mann–Whitney test. Categorical data were analyzed using Pearson’s Chi-Square test. Continuous variables with normal distribution are reported as mean ± standard deviation (SD), while those without normal distribution are reported as median (range). Categorical data are presented as percentages. Predictors of in-hospital new-onset atrial fibrillation were evaluated using logistic regression analysis. Variables were selected for multivariable models if they had *p* < 0.10 in univariate analysis or were supported by previous evidence (e.g., age, LA diameter, EF, hypertension), consistent with TRIPOD guidelines. For primary outcomes, MACE and all-cause mortality predictors were assessed using Cox regression modeling. To evaluate the role of SYNTAX, Clinical SYNTAX, CHA2DS2-VASc, and GRACE scores in predicting in-hospital new-onset atrial fibrillation, cut-off values were analyzed using receiver operating characteristic (ROC) curve analysis. Sensitivity and specificity calculations were performed where significant threshold values were found. A *p* value < 0.05 was considered statistically significant. Multivariable regression tables display all covariates entered into each model, including those that did not reach statistical significance, to provide transparency regarding model construction; these represent adjusted associations, and only variables with *p* < 0.05 were interpreted as independent predictors.

## 3. Results

### 3.1. Patient Characteristics and Follow-Up

A total of 979 patients diagnosed with NSTEMI who met the inclusion criteria were included in the final analysis. The demographic and clinical characteristics of the overall study population are presented in Table 1. Laboratory parameters revealed a hemoglobin level of 13.6 g/dL (7.6–19.7 g/dL), a leukocyte count of 9.3 (3.4–22.8), and a creatinine level of 0.89 mg/dL (0.39–3.5 mg/dL). The LDL cholesterol level was 123 mg/dL, with a total cholesterol level of 187 mg/dL. The median HbA1c level was 6.1% (4–16.4%), and the median troponin level was observed to be 0.179 ng/mL (0.014–10 ng/mL). The median follow-up duration for the included patients was 86 (IQR 36–100; range 0.1–144) months. Clinical follow-up data were available for 947 patients (96.7%), while 32 patients (3.3%) were lost to follow-up due to missing records.

Regarding the baseline angiographic characteristics, 104 (10.6%) patients had no significant stenosis, 388 (39.6%) had single-vessel disease, 278 (28.4%) had double-vessel disease, and 209 (21.3%) had triple-vessel disease, with a median SYNTAX score of 10 (0–46.5). After angiographic assessment, 537 (54.9%) patients underwent PCI, 193 (19.7%) underwent CABG surgery, and 249 (25.4%) were managed with medical follow-up based on angiographic and clinical evaluation.

### 3.2. Evaluation of New-Onset Atrial Fibrillation

Among the 979 patients included in the study, 80 (8.2%) were diagnosed with new-onset atrial fibrillation/flutter. When the patients were evaluated in two groups, their baseline demographic and clinical characteristics are presented in Table 1, with additional comparisons summarized below.

Examining the baseline angiographic characteristics and management strategies of patients, it was observed that among those with in-hospital new-onset atrial fibrillation (AF), the proportion of patients with multivessel disease was higher, whereas in patients without AF, there was a higher proportion of those with either no significant coronary artery disease or single-vessel disease (*p* < 0.001). Consistent with these findings, the proportion of patients undergoing coronary artery bypass grafting (CABG) was higher in the AF group, while the number of patients in the non-AF group receiving percutaneous coronary intervention (PCI) and medical management was greater (*p* < 0.001). Overall, the average SYNTAX score for patients with new-onset AF was 16 (2–33), whereas it was 10 (0–46.5) for those without, showing a significant difference (*p* < 0.001). It was observed that hemoglobin (12.9 vs. 13.6 g/dL), lymphocyte count (1.85 vs. 2.3 × 10^3^/mm^3^), and triglyceride levels (129 vs. 153 mg/dL) were significantly lower in the group with atrial fibrillation (AF) (*p* = 0.006, *p* < 0.001, *p* = 0.007, respectively). Additionally, the neutrophil-to-lymphocyte ratio and fasting blood glucose levels were found to be significantly higher in the AF group (*p* = 0.001, *p* = 0.025, respectively). In patients with in-hospital AF, the risk scores were significantly higher compared to those without in-hospital AF (*p* < 0.001) (Table 1).

The primary outcomes and their components were analyzed in a comparative manner between the general population and the groups with and without NOAF, and the detailed results are presented in Table 2.

Two separate models were used to identify independent predictors of in-hospital NOAF in NSTEMI patients, as shown in Table 3. The first model identified age (*p* < 0.001) and left atrial diameter (*p* < 0.001) as independent predictors of AF, while the SYNTAX score alone was found to be insufficient for predicting AF (*p* = 0.059). Due to the heterogeneity of the patient population, exploratory post hoc analyses were performed in two forms: one entering the SYNTAX score as a binary variable at the established low/intermediate tertile boundary (>22), and one excluding the 104 patients with a SYNTAX score of 0 (*n* = 875); neither analysis was pre-specified in our protocol or corrected for multiple comparisons, and results should be interpreted as hypothesis-generating. In these analyses, the SYNTAX score still did not predict in-hospital AF (*p* = 0.138, *p* = 0.280, respectively).

In the same model, when age and CrCl were excluded from the components of the Clinical SYNTAX score and the analysis was done using the Clinical SYNTAX score alone, it was found that left atrial diameter (*p* < 0.001), left ventricular diastolic dysfunction (LVDD) (*p* = 0.012), female sex (*p* = 0.007), and the Clinical SYNTAX score (*p* = 0.014) were parameters that could be used for independent prediction (Table 3).

The ROC curve analyses performed to evaluate the predictive power of risk scores for in-hospital NOAF are shown in Figure 2. All four scores discriminated in-hospital NOAF better than chance (all *p* < 0.001): the AUC was 0.724 (95% CI 0.673–0.775) for the Clinical SYNTAX score, 0.717 (0.663–0.772) for the CHA2DS2-VASc score, 0.701 (0.645–0.756) for the GRACE score, and 0.644 (0.588–0.699) for the SYNTAX score. In pairwise DeLong comparisons, the Clinical SYNTAX, CHA2DS2-VASc, and GRACE scores did not differ significantly from one another (all *p* > 0.5); the Clinical SYNTAX score discriminated NOAF significantly better than the anatomical SYNTAX score (difference 0.081, *p* < 0.001), whereas the CHA2DS2-VASc (*p* = 0.066) and GRACE (*p* = 0.15) scores did not differ significantly from it. In the predictive value analysis for detecting in-hospital new-onset atrial fibrillation using the Clinical SYNTAX score, a threshold value of Clinical SYNTAX ≥17.77 was observed to predict in-hospital atrial fibrillation with 75% sensitivity and 65% specificity.

Several additional sensitivity analyses were performed to address the modest events-per-variable ratio (6.7–8.0) of the NOAF logistic models. Firth’s penalized (bias-corrected) logistic regression confirmed that the Clinical SYNTAX score remained an independent predictor of NOAF (OR 1.009, 95% CI 1.002–1.016, *p* = 0.012), indicating this finding is robust to overfitting concerns. Calibration was satisfactory (Hosmer–Lemeshow χ^2^ = 6.25, df = 6, *p* = 0.396), and bootstrap resampling (200 iterations) showed only modest optimism (0.024), yielding an optimism-corrected AUC of 0.764 (apparent AUC 0.788). For the CSS ≥ 17.77 cutoff specifically, bootstrap resampling (500 iterations) confirmed stable discrimination (AUC 0.725, 95% CI 0.674–0.773) and stable sensitivity/specificity (75%/65%) at this threshold, although the optimal cutoff itself varied across resamples (95% CI 12.8–25.0), suggesting it should be treated as an approximate, internally derived threshold pending external validation.

Because CSS incorporates age, renal function, and EF, this univariate comparison does not isolate CSS’s incremental value over anatomical burden alone. To test this directly, we fit two non-overlapping multivariable logistic regression models with identical clinical covariates (male sex, LVDD, hypertension, hemoglobin, LDL, neutrophil-to-lymphocyte ratio, fasting blood glucose, and LA diameter): one retains the SYNTAX score together with age, CrCl, and EF as separate terms, and one replaces these four variables with CSS alone. The SYNTAX-score-based model discriminated NOAF significantly better than the CSS-based model (AUC 0.831, 95% CI 0.787–0.874, vs. 0.788, 95% CI 0.739–0.836; DeLong test, difference = −0.043, 95% CI −0.071 to −0.014, *p* = 0.003), indicating that modeling age, renal function, and EF as separate covariates alongside the SYNTAX score captures their prognostic information more efficiently than combining them into a single composite score.

Given that CABG, PCI, and medically managed patients may differ in the underlying mechanism of NOAF, a treatment-stratified sensitivity analysis was performed. In-hospital NOAF incidence differed by revascularization strategy: 15.0% (29/193) after CABG, 6.0% (32/537) after PCI, and 7.6% (19/249) with medical therapy. A formal likelihood-ratio test for interaction between the Clinical SYNTAX score and treatment strategy was significant (χ^2^ = 16.24, df = 2, *p* < 0.001), indicating that the association between CSS and NOAF is not uniform across strategies. CSS discriminated NOAF status within the PCI subgroup (median CSS 21.7 vs. 9.9 in NOAF(+) vs. NOAF(−), *p* < 0.001) but not within the CABG subgroup (median CSS 36.4 vs. 36.4, *p* = 0.79), where CSS was uniformly elevated regardless of NOAF status, consistent with CABG candidacy itself requiring a high anatomical burden.

Given this treatment-dependent heterogeneity, we designate the PCI- and medically managed population (*n* = 786) as the primary analysis population for the CSS–NOAF relationship. Within this population, the non-overlapping model comparison described above yielded an AUC of 0.861 (95% CI 0.809–0.913) for the SYNTAX-score-based model versus 0.821 (95% CI 0.765–0.877) for the CSS-based model (DeLong test, difference = −0.040, 95% CI −0.071 to −0.010, *p* = 0.009), consistent with the pooled-cohort findings above. CSS remained an independent predictor of NOAF within its own model in this population (OR 1.010, 95% CI 1.001–1.019, *p* = 0.028).

In contrast, among CABG-managed patients (*n* = 193; NOAF incidence 15.0%), CSS showed no discriminative ability for NOAF (AUC 0.515, 95% CI 0.406–0.625), consistent with the near-identical median CSS between NOAF-positive and NOAF-negative patients reported above (36.4 vs. 36.4, *p* = 0.79). CSS should therefore not be used to predict NOAF risk in CABG-managed patients; post-CABG AF likely arises through mechanisms distinct from the anatomical and clinical burden captured by CSS and warrants separate study.

### 3.3. Evaluation for All-Cause Mortality

To identify independent predictors of all-cause mortality as a primary endpoint, Cox regression analysis was performed, including variables found to be significant in univariate analyses. The regression analysis revealed that in-hospital atrial fibrillation (*p* = 0.035), older age (*p* < 0.001), hypertension (*p* = 0.010), low CrCl (*p* = 0.002), and EF < 60% (*p* = 0.004) were independent parameters associated with long-term all-cause mortality in NSTEMI patients. However, the SYNTAX score and GRACE score above 140, as well as atrial fibrillation within the first 12 months of follow-up, were not found to be significant independent predictors of long-term mortality (*p* = 0.059, *p* = 0.288, *p* = 0.065, respectively) (Table 4 Model 1). When the Clinical SYNTAX score, which showed correlation with the SYNTAX score, was used instead of the SYNTAX score, it was found to significantly predict all-cause mortality (*p* = 0.04), with other significant variables maintaining their significance. The continued significance of the Clinical SYNTAX score, when used alongside the SYNTAX score, is shown in Model 2 in Table 4 (*p* = 0.013, *p* = 0.794, respectively).

Because CSS is a function of age, CrCl, and EF, its coefficient in Model 2 above is estimated while these same variables remain in the model, and a VIF < 2 does not rule out this conceptual overlap. We therefore compared two non-overlapping Cox models sharing identical clinical covariates (in-hospital NOAF, LVDD, hypertension, previous MI, smoking, male sex, LDL, HDL, GRACE ≥ 140, anemia, and AF at 12-month follow-up): one retained the SYNTAX score together with age, CrCl, and EF < 60% as separate covariates, and one replaced these four variables with CSS alone. Harrell’s C-index was 0.752 for the SYNTAX-score-based model versus 0.730 for the CSS-based model; bootstrap resampling (2000 iterations) confirmed this difference was significant (difference = −0.022, 95% CI −0.038 to −0.006, *p* = 0.0065), indicating that the SYNTAX-score-based model discriminated long-term mortality significantly better than CSS alone.

Because AF within the first 12 months can only be ascertained in patients who survive long enough for it to be observed, modeling it as a fixed baseline covariate risks immortal-time bias. To address this, we performed a 12-month landmark analysis, restricting to patients event-free at 12 months (*n* = 877; 239 subsequent deaths) and re-fitting the model with follow-up time reset to the landmark. AF within the first 12 months remained associated with all-cause mortality at a similar magnitude (HR 1.804, 95% CI 0.955–3.409, *p* = 0.069, versus HR 1.712, 95% CI 0.975–3.005, *p* = 0.061 in the original fixed-covariate model), indicating the original estimate was not materially distorted by immortal-time bias in this cohort.

Due to the heterogeneity of the patient population and the high number of patients with SYNTAX scores < 22 (75.7%), an exploratory post hoc analysis was conducted using the established SYNTAX low/intermediate tertile boundary (>22); this cutoff is literature-based rather than data-derived, but the analysis itself was not pre-specified in our protocol. In this analysis, a SYNTAX score > 22 emerged as an independent predictor of all-cause mortality (HR = 1.351, *p* = 0.018, 95% CI 1.053–1.734). Additionally, in a further exploratory analysis excluding patients with a SYNTAX score of 0, using the same parameters in the 875 patients, the SYNTAX score was also identified as an independent predictor of all-cause mortality (HR = 1.011, *p* = 0.048, 95% CI 1.000–1.023). These post hoc findings were not corrected for multiple comparisons and should be considered hypothesis-generating rather than confirmatory.

### 3.4. Evaluation of Patients for MACEs (Death/MI/Stroke)

Baseline angiographic characteristics and revascularization strategies in patient groups with and without MACEs are shown in Table 5. To identify the independent predictors of the composite primary endpoint of death/MI/stroke, Cox regression analyses were performed in two separate models, including variables found significant in univariate analyses (Table 6 Model 1). The analysis identified NOAF (*p* = 0.034), EF < 60% (*p* = 0.032), hypertension (*p* = 0.019), age (*p* = 0.002), detection of atrial fibrillation within the first 12 months (*p* = 0.002), and Clinical SYNTAX score (*p* = 0.026) as independent predictors, while a GRACE score > 140 was not found to be an independent predictor (*p* = 0.950). In a separate analysis with the correlated SYNTAX score, SYNTAX was not identified as an independent predictor (*p* = 0.178) (Table 6 Model 2). Due to the heterogeneity of the patient population, in an exploratory post hoc analysis using the established >22 cutoff, a SYNTAX score > 22 was not found to be an independent predictor for the composite primary endpoint of death/MI/stroke (*p* = 0.126, 95% CI 0.953–1.478).

The same non-overlapping comparison applied to the composite MACE endpoint: Harrell’s C-index was 0.673 for the SYNTAX-score-based model versus 0.666 for the CSS-based model, a difference that did not reach statistical significance (bootstrap resampling, 2000 iterations; difference = −0.008, 95% CI −0.022 to 0.006, *p* = 0.28).

The same 12-month landmark approach used for mortality was applied to MACEs, restricting to patients event-free at 12 months (*n* = 841; 318 subsequent events). AF within the first 12 months remained associated with MACEs at a similar magnitude (HR 2.272, 95% CI 1.295–3.986, *p* = 0.004, versus HR 2.165, 95% CI 1.337–3.505, *p* = 0.002 in the original fixed-covariate model), again indicating that immortal-time bias did not materially distort this estimate.

Due to the correlation of parameters such as hypertension, gender, age, and previous MI with the CHA2DS2-VASc score in the model, an exploratory post hoc analysis excluding age was performed. This analysis demonstrated that the CHA2DS2-VASc score is an independent predictor for both all-cause mortality and the composite primary endpoint of death/MI/stroke (*p* = 0.001 and *p* = 0.002, respectively); this exploratory model, which was not a pre-specified primary analysis, is presented in Table 7.

## 4. Discussion

In this retrospective cohort of 979 NSTEMI patients followed for a median of 86 months, in-hospital NOAF occurred in 8.2% and was an independent predictor of long-term all-cause mortality and of the composite of death, MI, and stroke. The Clinical SYNTAX score was independently associated with NOAF, mortality, and MACEs, but non-overlapping model comparisons showed that its apparent advantage over the anatomical SYNTAX score reflects the prognostic weight of its clinical components (age, renal function, and ejection fraction) rather than incremental value of the composite. The CSS–NOAF association was confined to PCI- and medically managed patients and was absent after CABG.

The incidence of new-onset AF in acute coronary syndrome (ACS) has declined from 5–23% to 7–10% with advances in ACS management and earlier revascularization [21]. Our in-hospital NOAF incidence of 8.2% is lower than that reported in the TRACE registry (21% AF/flutter) [22], the OACIS study (12% among PCI-treated ACS patients) [4], the cohort of Braga et al. (10.1%) [23], and the NSTEMI-PCI series of Karasu et al. (12.5%) [24], most plausibly reflecting our exclusion of STEMI, prior AF, and valvular disease and our restriction to angiographically evaluated patients. The predictor profile overlaps substantially with these reports: as in OACIS and Braga et al., NOAF patients in our cohort were older and had larger left atria and lower ejection fraction, and, as in Karasu et al., the anatomical SYNTAX score was not an independent predictor. Independent predictors of in-hospital NOAF in our multivariable models were age, female sex, LA diameter, LVDD, and CSS (Table 3); hypertension and renal function differed between groups in univariable comparisons but did not retain independent significance, and the neutrophil-to-lymphocyte ratio was not significant in multivariable analysis. The association between coronary disease burden and NOAF is biologically plausible: advanced atherosclerosis and impaired perfusion raise left atrial pressure, promote atrial stretch and ischemia-related autonomic imbalance, and drive systemic inflammation, each contributing to atrial remodeling and arrhythmogenesis [25,26].

Although CSS, but not the anatomical SYNTAX score (*p* = 0.059), was independently associated with NOAF, and this association was robust to penalized (Firth) regression, calibration testing, and bootstrap validation (Section 3.2), independence does not establish incremental value. Because CSS is a fixed combination of the SYNTAX score with age, renal function, and EF, a model entering these four variables as separate terms discriminated NOAF significantly better than CSS alone (Section 3.2); the higher univariate AUC of CSS over SS therefore reflects the contribution of its clinical constituents rather than added value of the composite itself, and consistent with this, the CHA2DS2-VASc and GRACE scores, which likewise incorporate age and clinical variables, discriminated NOAF as well as CSS in univariate comparison (Section 3.2). The CSS ≥ 17.77 threshold, whose optimal value varied across bootstrap resamples, should likewise be regarded as an approximate, internally derived cutoff pending external validation.

The treatment strategy modifies the CSS–NOAF relationship. In the CABG series of Oktay et al. [27], postoperative AF occurred in 31.7% and both SS and CSS were independent predictors; in our cohort NOAF after CABG was less frequent (15.0%), likely reflecting differences in monitoring intensity and ECG frequency, and the CSS × treatment interaction was significant (*p* < 0.001). CSS discriminated NOAF among PCI- and medically managed patients but not among CABG-managed patients, in whom CSS was uniformly elevated regardless of NOAF status and had no discriminative ability (AUC 0.515), consistent with post-CABG AF arising through mechanisms distinct from atherosclerotic burden, such as cardiopulmonary bypass, atriotomy, and the surgical inflammatory response. We therefore regard the PCI- and medically managed population as the primary population for the CSS–NOAF relationship; within it, CSS remained an independent predictor of NOAF and the SS-based non-overlapping model again discriminated better than the CSS-based model (Section 3.2). CSS should not be used to predict NOAF risk in CABG-managed patients.

Consistent with TRACE [22] and the CREDO-Kyoto AMI registry [28], in which in-hospital NOAF was associated with higher in-hospital and long-term mortality, NOAF patients in our cohort had higher in-hospital mortality (5% vs. 1.6%), and NOAF was an independent predictor of both long-term all-cause mortality and the composite of death, MI, and stroke (Table 4 and Table 6). Not all studies agree: Goldberg et al. [29] found that the univariable association between NOAF and mortality disappeared after adjustment for heart failure and cardiogenic shock, a discrepancy that may reflect their indirect ascertainment of AF, limited revascularization data, and a predominantly STEMI population. In the cohort of Hofer et al., monitored with 72 h telemetry [30], NOAF was detected in 10.9% and carried a 63.4% long-term cardiovascular mortality; our lower incidence (8.2%) is consistent with the absence of continuous telemetry beyond the first 24 h (a potential surveillance limitation), yet NOAF remained an independent predictor of mortality.

Anticoagulation after NOAF in ACS remains a treatment gap. In CREDO-Kyoto only 28% of NOAF patients were discharged on oral anticoagulation and 23.7% received triple antithrombotic therapy [28]. ACC/AHA guidelines recommend anticoagulation for AF with CHA2DS2-VASc ≥ 2 but give no specific guidance for in-hospital NOAF, whereas the 2023 ESC guidelines for acute coronary syndromes give a class IIa recommendation to consider long-term oral anticoagulation, preferably with a NOAC, in patients with de novo AF during the acute phase of ACS, depending on the CHA2DS2-VASc score and after weighing bleeding risk (HAS-BLED) and the need for concomitant antiplatelet therapy [8,31]. Hofer et al. reported lower mortality with triple but not dual antithrombotic therapy [30]; we could not assess this in our cohort. Oral anticoagulation was prescribed in 42.5% of our NOAF patients (Table 1), and the higher rate of ischemic stroke among NOAF patients during follow-up (10% vs. 2.1%, Table 2) argues for individualized post-discharge anticoagulation decisions weighing thromboembolic and bleeding risk, although our event numbers were too small to evaluate the effect of anticoagulation on outcomes.

Regarding long-term outcomes, in the ARTS II study CSS but not SS was an independent predictor of adverse events in patients with multivessel disease [20], and in the SIRTAX substudy CSS outperformed SS for 5-year mortality with a smaller advantage for MACE [32]. Our primary models show a similar pattern: CSS was an independent predictor of all-cause mortality and MACEs, whereas SS did not reach significance in either model (Table 4 and Table 6). As for NOAF, however, this pattern largely reflects the prognostic weight of age, renal function, and EF embedded within CSS: in non-overlapping models with identical clinical covariates, the SS-based model with these variables as separate terms discriminated mortality significantly better than the CSS-based model and did not differ significantly for MACEs (Section 3.3 and Section 3.4). Our findings therefore support CSS as a convenient single composite that retains independent prognostic association, but not as superior to SS once SS is considered together with its own clinical correlates. The weaker performance of the anatomical SYNTAX score in our cohort compared with the PCI-only chronic coronary syndrome population of SIRTAX may also reflect our inclusion of patients with non-obstructive disease (SYNTAX score 0, 10.6%) and of CABG- and medically managed patients; the exploratory finding that SS > 22 predicted mortality but not MACEs should be regarded as hypothesis-generating (Section 3.3).

Clinically, these findings identify in-hospital NOAF as a marker of substantially elevated long-term risk in NSTEMI, warranting attention to rhythm surveillance during admission and to individualized anticoagulation decisions at discharge. CSS offers a convenient single-number summary of anatomical and clinical burden, but clinicians and future risk models should not expect it to outperform direct consideration of the SYNTAX score together with age, renal function, and ejection fraction, and it should not be applied to CABG-managed patients. External validation in prospective cohorts with continuous rhythm monitoring and time-resolved AF ascertainment is needed before any CSS threshold is used to guide management.

## 5. Limitations

1. The retrospective nature of the study limited the ability to adequately monitor patients’ medication adherence and follow-up on their medical treatments throughout the study period, thereby preventing an assessment of the impact of medical therapy on outcomes.
2. The effectiveness of the SYNTAX score is primarily validated in patients with multivessel disease and is calculated in those with significant stenosis. In our study, 10.6% of the population had no significant stenosis, resulting in a SYNTAX score of 0, which could affect the score’s effectiveness; in patients presenting with a totally occluded infarct-related artery, baseline SYNTAX scoring may also under-assess lesion complexity distal to the occlusion.
3. There was a possibility of including patients with undiagnosed asymptomatic paroxysmal AF who may have been incidentally identified during their index hospitalization for AMI, which might have impacted the study population.
4. The study’s start date in 2011 means that some patient groups did not have access to newer antiplatelet agents (prasugrel, ticagrelor), which could have influenced outcomes.
5. Current guidelines recommend the use of novel oral anticoagulants (NOACs) over warfarin in AF patients due to their positive impact on outcomes. However, among NOAF patients receiving anticoagulation in our study, only 5 of 34 (14.7%) received a NOAC rather than warfarin.
6. The early invasive treatment strategies for acute coronary syndrome during the initial period of our study may differ from current guideline recommendations, potentially affecting outcomes.
7. The study period coincided with the COVID-19 pandemic, which may have led to an increase in mortality due to some deaths being related to COVID-19, potentially impacting the overall mortality rate.
8. The association of the CHA2DS2-VASc and GRACE scores with NOAF may partly reflect shared underlying risk factors, such as age, rather than mechanisms specific to each score; although the univariate discrimination of the four scores was compared directly (Section 3.2), a formal incremental predictive value analysis of CSS against these scores in multivariable models (e.g., net reclassification or integrated discrimination improvement) was not performed and is recommended for future prospective studies.
9. Long-term follow-up combined national and hospital records. Three modeling limitations were addressed by sensitivity analyses rather than eliminated: (i) AF within the first 12 months was modeled as a fixed covariate, risking immortal-time bias; a 12-month landmark analysis (Section 3.3 and Section 3.4) showed that its association with mortality and MACEs was not materially altered. (ii) The NOAF logistic models had a modest events-per-variable ratio (6.7–8.0); Firth’s penalized regression, calibration, and bootstrap validation (Section 3.2) confirmed that the core findings were robust. (iii) In the primary models, CSS was retained alongside some of its own components; a VIF < 2 excluded severe collinearity but not conceptual overlap, so non-overlapping models were fitted and compared directly (Section 3.2, Section 3.3 and Section 3.4). Because the SYNTAX-score-based comparators contain three additional free parameters, part of their apparent advantage may reflect model size, and all estimates are apparent (in-sample) rather than externally validated.
10. Formal inter-observer reproducibility of SYNTAX scoring was not quantified, and different lengths of stay between NOAF groups raise the possibility of surveillance bias. In addition, the predictive value of CSS for NOAF varies by revascularization strategy, with reduced utility in CABG-managed patients specifically; this treatment-stratified analysis is detailed in the Discussion.

## 6. Conclusions

In NSTEMI patients, long-term mortality rates are observed to be high due to accompanying comorbidities. New-onset in-hospital atrial fibrillation, independent of these comorbidities, has been shown to be an independent predictor of long-term prognosis, affecting both all-cause mortality and the primary composite endpoint of death/MI/stroke.

The Clinical SYNTAX score, which combines anatomical assessment of coronary artery disease with clinical parameters, remained an independent predictor of all-cause mortality and the composite endpoint of death/MI/stroke across the entire NSTEMI population; however, when compared directly against the SYNTAX score in non-overlapping models, it did not demonstrate superior discrimination for these endpoints, and a model retaining the SYNTAX score alongside age, renal function, and EF as separate covariates performed at least as well. Additionally, factors directly associated with these endpoints—such as age, left atrial diameter, female gender, and left ventricular diastolic dysfunction—along with the Clinical SYNTAX score have been shown to be independent predictors of in-hospital atrial fibrillation. In univariate ROC comparison, the Clinical SYNTAX, CHA2DS2-VASc, and GRACE scores discriminated in-hospital NOAF comparably, all numerically better than the anatomical SYNTAX score alone, although only the difference between the Clinical SYNTAX and SYNTAX scores reached statistical significance. Moreover, while our study did not demonstrate the effectiveness of oral anticoagulation (OAC) in reducing stroke due to the low number of events, the high rate of stroke in patients with new-onset AF during long-term follow-up suggests that, in line with other studies, OAC use should be considered based on the patient’s bleeding and ischemic stroke risk.

In conclusion, given the pathophysiology of NSTEMI patients and the high prevalence of comorbidities, incorporating clinical factors alongside coronary anatomy—whether as a combined composite score or as separate covariates—appears more informative than relying on coronary anatomy alone for predicting both long-term outcomes and in-hospital new-onset atrial fibrillation; further prospective studies with formal incremental-value testing are warranted to clarify the optimal approach to combining these variables.

## Figures and Tables

**Figure 1:**
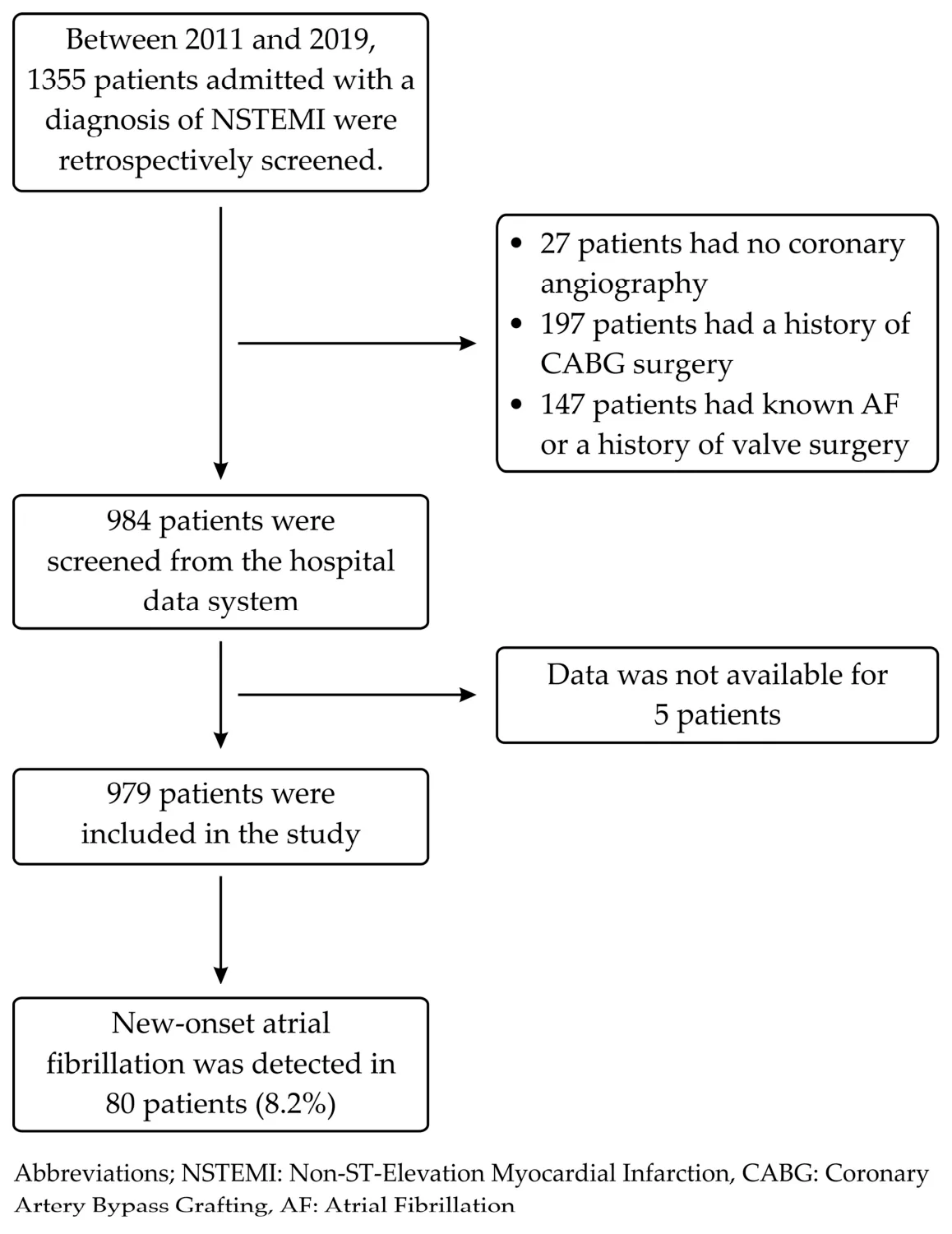
Study flowchart.

**Figure 2:**
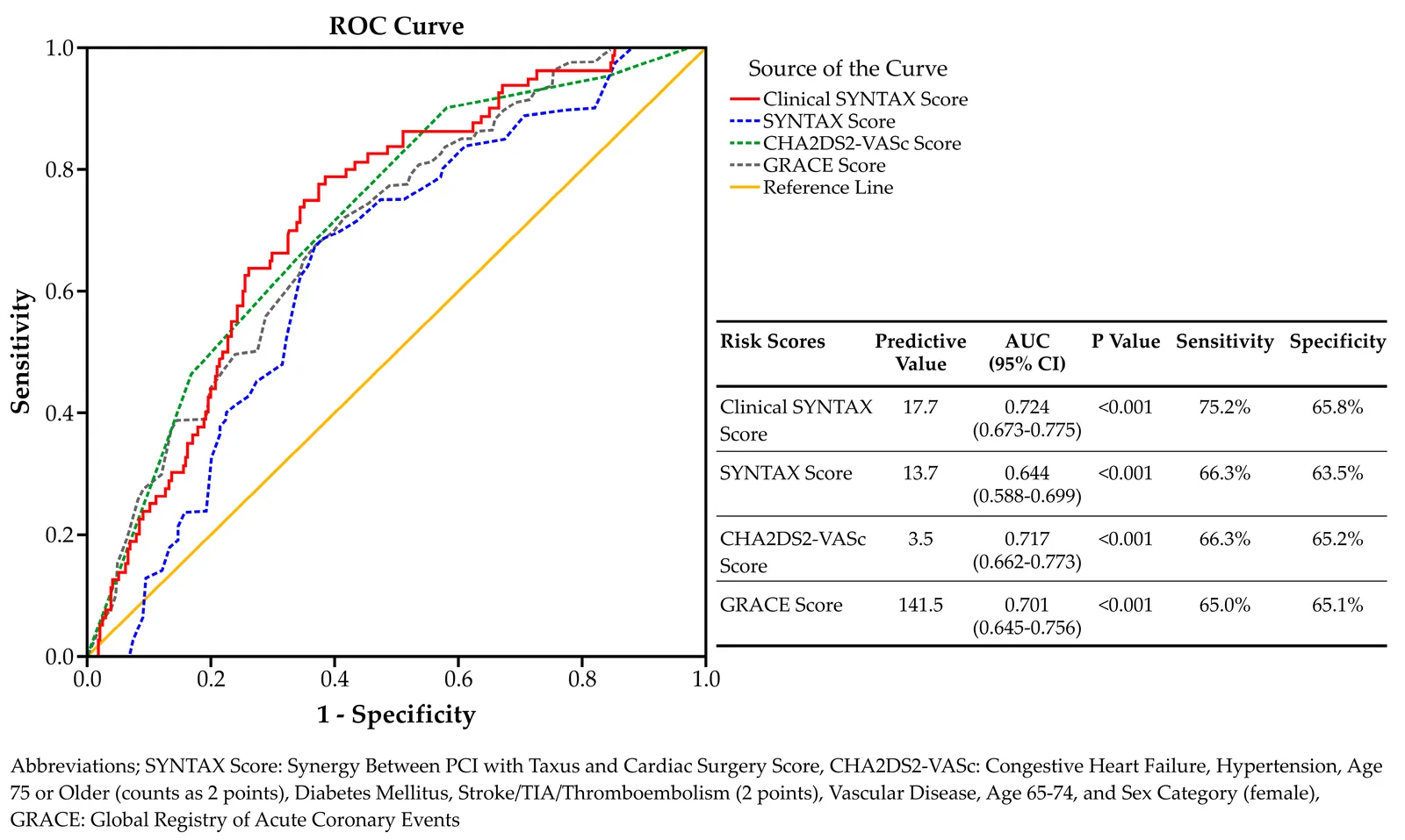
ROC curve analysis conducted to evaluate the predictive power of risk scores in forecasting NOAF (entire cohort, *n* = 979; the corresponding non-overlapping model comparison in the primary PCI/medically managed population is reported in the text).

**Table 1:** The baseline demographic and clinical characteristics of the whole population and the groups with and without NOAF.

Variables	Total, *n* = 979	NOAF (+), *n* = 80 (8.2%)	NOAF (−), *n* = 899 (91.8%)	*p* Value
Age, (years) *	59 (24–92)	71 (44–86)	59 (24–92)	<0.001
Male, *n* (%)	698 (71.3)	44 (55)	654 (72.7)	0.001
Previous MI, *n* (%)	198 (20.2)	18 (22.5)	180 (20)	0.597
Previous PCI, *n* (%)	262 (26.8)	25 (31.3)	237 (26.4)	0.344
HT, *n* (%)	572 (58.4)	58 (72.5)	514 (57.2)	0.008
DM, *n* (%)	351 (35.9)	33 (41.3)	318 (35.4)	0.294
Medications used at discharge, *n* (%)				
ASA	928 (94.8)	74 (92.5)	854 (95)	0.336
Clopidogrel	466 (47.6)	43 (53.8)	423 (47.1)	n/a
Ticagrelor	266 (27.2)	4 (5)	262 (29.1)	n/a
P2Y12 inhibitor	737 (75.3)	47 (58.8)	690 (76.8)	<0.001
DAPT	706 (72.1)	42 (52.5)	664 (73.9)	<0.001
Beta blocker	895 (91.4)	75 (93.8)	820 (91.2)	0.437
RAAS inhibitor	845 (86.3)	68 (85)	777 (86.4)	0.722
Statin	867 (88.6)	65 (81.3)	802 (89.2)	0.032
Oral anticoagulant therapy	86 (8.8)	34 (42.5)	52 (5.8)	<0.001
Triple antithrombotic therapy (TAT)	57 (5.8)	19 (23.8)	38 (4.2)	<0.001
In-hospital arrhythmia, *n* (%)	120 (12.3)	80 (100)	40 (4.3)	n/a
Ventricular arrhythmia, *n* (%)	45 (4.6)	6 (7.5)	39 (4.3)	n/a
12-month follow-up atrial fibrillation, *n* (%)	23 (2.3)	n/a	n/a	n/a
Length of stay (days) *	5 (1–45)	7 (2–45)	5 (1–45)	<0.001
ACEF score *	1.13 (0.40–6.62)	1.6 (0.7–5.8)	1.1 (0.4–6.6)	<0.001
SYNTAX score *	10 (0–46.5)	16 (2–33)	10 (0–46.5)	<0.001
Clinical SYNTAX score *	12.3 (0–230)	27.5 (2.5–124)	11.3 (0–230)	<0.001
CHA2DS2-VASc score *	3 (0–7)	4 (1–7)	3 (0–7)	<0.001
GRACE score *	131 (31–260)	153 (92–260)	128 (31–248)	<0.001
LA diameter (mm) *	38 (23–63)	43 (32–54)	38 (23–63)	<0.001
LA ≥ 40 mm, *n* (%)	388 (39.6)	54 (67.5)	334 (37.2)	<0.001
LVDD, *n* (%)	537 (54.9)	62 (77.5)	475 (52.8)	<0.001
EF (%) *	60 (18–60)	50 (25–60)	60 (18–60)	0.010
EF ≤ 40%, *n* (%)	123 (12.6)	17 (21.3)	106 (11.8)	0.014
EF < 60%, *n* (%)	469 (47.9)	47 (58.8)	422 (46.9)	0.043
CrCl ≥ 90, *n* (%)	499 (51)	25 (31.3)	474 (52.7)	<0.001
CrCl: 60–89, *n* (%)	343 (35)	27 (33.8)	316 (35.2)	0.801
CrCl < 60, *n* (%)	137 (14)	28 (35)	109 (12.1)	<0.001

Abbreviations: MI: myocardial infarction; PCI: percutaneous coronary intervention; HT: hypertension; DM: diabetes mellitus; ASA: aspirin; DAPT: dual antiplatelet therapy; RAAS Inhibitor: renin-angiotensin-aldosterone system inhibitor; SYNTAX Score: Synergy Between PCI with Taxus and Cardiac Surgery Score; CHA2DS2-VASc: congestive heart failure, hypertension, age 75 or older (counts as 2 points), diabetes mellitus, stroke/TIA/thromboembolism (2 points), vascular disease, age 65–74, and sex category (female); GRACE: Global Registry of Acute Coronary Events; LA: left atrium; LVDD: left ventricular diastolic dysfunction; EF: ejection fraction; CrCl: creatinine clearance (Cockcroft–Gault). Triple antithrombotic therapy was defined as concurrent prescription of aspirin, a P2Y12 inhibitor, and an oral anticoagulant. * Median (minimum–maximum).

**Table 2:** The primary outcomes and their components analyzed in a comparative manner between the general population and the groups with and without NOAF.

Primary Endpoints	General Population	NOAF (+), *n* = 80 (8.2%)	NOAF (−), *n* = 899 (91.8%)	*p* Value
Death, *n* (%)	297 (30.3)	48 (60)	249 (27.7)	**<0.001**
In-hospital death	18 (1.8)	4 (5)	14 (1.6)	**0.028**
MI, *n* (%)	168 (17.2)	11 (13.8)	157 (17.5)	0.399
Ischemic stroke, *n* (%)	27 (2.8)	8 (10)	19 (2.1)	**<0.001**
MACEs (death/MI/stroke), *n* (%)	412 (42.1)	53 (66.3)	359 (39.9)	**<0.001**
Cardiovascular hospitalization (%)	276 (28.2)	33 (41.3)	243 (27)	**0.007**

Abbreviations: MI: myocardial infarction, MACEs: major adverse cardiovascular events (death/myocardial infarction/ischemic stroke). Bold values indicate statistical significance (*p* < 0.05).

**Table 3:** Two separate regression analyses conducted to identify independent predictors of NOAF.

Independent Predictors Model 1	OR	95% CI	*p* Value	Independent Predictors Model 2	OR	95% CI	*p* Value
Male sex	0.581	0.323–1.042	0.069	Male sex	0.467	0.267–0.816	**0.007**
LVDD	1.790	0.986–3.248	0.056	LVDD	2.120	1.182–3.802	**0.012**
HT	0.789	0.431–1.446	0.444	HT	0.952	0.530–1.709	0.869
Hemoglobin	1.092	0.941–1.267	0.244	Hemoglobin	1.050	0.907–1.217	0.512
SYNTAX score	1.022	0.999–1.045	0.059	Clinical SYNTAX score	1.009	1.002–1.016	**0.014**
LDL	0.999	0.993–1.005	0.786	LDL	0.998	0.992–1.004	0.551
Age	1.077	1.043–1.113	**<0.001**				
Neutrophil-to-lymphocyte ratio	1.033	0.990–1.077	0.135	Neutrophil-to-lymphocyte ratio	1.023	0.984–1.063	0.253
EF	1.006	0.980–1.032	0.669	EF	1.011	0.985–1.038	0.421
Fasting blood glucose	1.004	1.000–1.007	0.070	Fasting blood glucose	1.002	0.999–1.006	0.216
LA diameter	1.126	1.072–1.184	**<0.001**	LA diameter	1.144	1.091–1.200	**<0.001**
CrCl	1.004	0.992–1.016	0.486				

Abbreviations: LVDD: left ventricular diastolic dysfunction, HT: hypertension, SYNTAX Score: Synergy Between PCI with Taxus and Cardiac Surgery Score, LDL: low-density lipoprotein, EF: ejection fraction, LA: left atrium. Bold values indicate statistical significance (*p* < 0.05).

**Table 4:** Two separate Cox regression models conducted to identify independent predictors of all-cause mortality.

Independent Predictors (Model 1)	HR	95% CI	*p* Value	Independent Predictors (Model 2)	HR	95% CI	*p* Value
New-onset atrial fibrillation	1.421	1.024–1.971	**0.035**	New-onset atrial fibrillation (NOAF)	1.464	1.054–2.034	**0.023**
LVDD	1.269	0.968–1.664	0.085	LVDD	1.262	0.961–1.656	0.094
HT	1.463	1.097–1.950	**0.010**	HT	1.445	1.082–1.930	**0.013**
Previous MI	1.146	0.869–1.511	0.334	Previous MI	1.105	0.835–1.462	0.484
Smoking history	1.164	0.895–1.513	0.259	Smoking history	1.167	0.897–1.516	0.250
Male sex	1.184	0.898–1.562	0.232	Male sex	1.199	0.910–1.580	0.197
LDL	1.001	0.998–1.003	0.739	LDL	1.001	0.998–1.004	0.542
Age	1.037	1.021–1.054	**<0.001**	Age	1.035	1.018–1.051	**<0.001**
HDL	1.002	0.992–1.013	0.672	HDL	1.002	0.992–1.013	0.665
SYNTAX score	1.010	1.000–1.021	0.059	SYNTAX score	0.998	0.982–1.014	0.794
EF < 60%	1.439	1.124–1.842	**0.004**	EF < 60%	1.398	1.089–1.794	**0.009**
GRACE score > 140	1.157	0.884–1.515	0.288	GRACE score > 140	1.157	0.882–1.518	0.292
CrCl	0.990	0.985–0.996	**0.002**	CrCl	0.993	0.987–0.999	**0.021**
Anemia	1.259	0.968–1.612	0.068	Anemia	1.268	0.990–1.625	0.060
NOAF at 12-month follow-up	1.700	0.968–2.984	0.065	NOAF at 12-month follow-up	1.723	0.980–3.030	0.059
				Clinical SYNTAX score	1.006	1.001–1.011	**0.013**

Abbreviations: LVDD: left ventricular diastolic dysfunction, HT: hypertension, MI: myocardial infarction, LDL: low-density lipoprotein, HDL: high-density lipoprotein, SYNTAX score: Synergy Between PCI with Taxus and Cardiac Surgery Score, EF: ejection fraction, GRACE: Global Registry of Acute Coronary Events, CrCl: creatinine clearance (Cockcroft–Gault), NOAF: new-onset atrial fibrillation. Bold values indicate statistical significance (*p* < 0.05).

**Table 5:** Baseline angiographic characteristics and revascularization strategies in patient groups with and without MACEs.

Variables	MACEs (+), *n* = 412 (42.1%)	MACEs (−), *n* = 567 (57.9%)	*p* Value
Number of diseased vessels (≥50% stenosis), *n* (%)			
No disease	27 (6.6)	77 (13.6)	**<0.001**
Single-vessel disease	142 (34.5)	246 (43.4)	
Two-vessel disease	128 (31.1)	150 (26.5)	
Three-vessel disease	115 (27.9)	94 (16.6)	
Revascularization strategy, *n* (%)			
Medical management	114 (27.7)	135 (23.8)	0.303
PCI	215 (52.2)	322 (56.8)	
CABG	83 (20.1)	110 (19.4)	
SYNTAX score *	12 (0–46.5)	9 (0–45)	**<0.001**

Abbreviations: MACEs: major adverse cardiovascular events (death/myocardial infarction/ischemic stroke), PCI: percutaneous coronary intervention, CABG: coronary artery bypass grafting, SYNTAX Score: Synergy Between PCI with Taxus and Cardiac Surgery Score. * Median (minimum–maximum). Bold values indicate statistical significance (*p* < 0.05).

**Table 6:** Two separate Cox regression models conducted to identify independent predictors of MACEs (death/MI/stroke).

Independent Predictors (Model 1)	HR	95% CI	*p* Value	Independent Predictors (Model 2)	HR	95% CI	*p* Value
NOAF	1.389	1.025–1.883	**0.034**	NOAF	1.371	1.012–1.858	**0.042**
LVDD	1.189	0.950–1.489	0.130	LVDD	1.188	0.949–1.486	0.133
HT	1.327	1.048–1.680	**0.019**	HT	1.336	1.056–1.690	**0.016**
Previous MI	1.232	0.972–1.560	0.084	Previous MI	1.252	0.989–1.585	0.062
Smoking history	1.059	0.850–1.319	0.609	Smoking history	1.056	0.847–1.316	0.627
Male sex	1.080	0.850–1.374	0.528	Male sex	1.077	0.846–1.372	0.546
LDL	1.000	0.998–1.003	0.727	LDL	1.000	0.998–1.003	0.861
Age	1.021	1.008–1.035	**0.002**	Age	1.023	1.009–1.036	**0.001**
HDL	0.995	0.986–1.004	0.300	HDL	0.995	0.986–1.004	0.291
Clinical SYNTAX score	1.004	1.000–1.007	**0.026**	SYNTAX score	1.006	0.997–1.016	0.178
EF < 60%	1.254	1.019–1.543	**0.032**	EF < 60%	1.282	1.043–1.575	**0.018**
GRACE score > 140	1.007	0.802–1.265	0.950	GRACE score > 140	1.011	0.806–1.268	0.926
CrCl	0.996	0.991–1.001	0.105	CrCl	0.995	0.991–1.000	**0.033**
Anemia	1.201	0.967–1.491	0.097	Anemia	1.203	0.970–1.494	0.093
NOAF at 12-month follow-up	2.154	1.331–3.485	**0.002**	NOAF at 12-month follow-up	2.149	1.328–3.479	**0.002**

Abbreviations: LVDD: left ventricular diastolic dysfunction, HT: hypertension, MI: myocardial infarction, LDL: low-density lipoprotein, HDL: high-density lipoprotein, SYNTAX Score: Synergy Between PCI with Taxus and Cardiac Surgery Score, EF: ejection fraction, GRACE: Global Registry of Acute Coronary Events, CrCl: creatinine clearance (Cockcroft–Gault), NOAF: new-onset atrial fibrillation. Bold values indicate statistical significance (*p* < 0.05).

**Table 7:** Exploratory Cox regression models substituting the CHA_2_DS_2_-VASc score for age, given the correlation between age and CHA_2_DS_2_-VASc score in the primary models.

Independent Predictors (Mortality)	HR	95% CI	*p* Value	Independent Predictors (MACEs)	HR	95% CI	*p* Value
New-onset atrial fibrillation	1.500	1.082–2.080	**0.015**	New-onset atrial fibrillation (NOAF)	1.439	1.063–1.947	**0.019**
LVDD	1.284	0.978–1.686	0.072	LVDD	1.203	0.961–1.505	0.107
HT	1.084	0.773–1.520	0.641	HT	1.061	0.800–1.408	0.681
Previous MI	1.103	0.836–1.454	0.489	Previous MI	1.205	0.952–1.526	0.121
Smoking history	1.138	0.876–1.477	0.333	Smoking history	1.052	0.845–1.308	0.651
Male sex	1.605	1.152–2.236	**0.005**	Male sex	1.359	1.021–1.811	**0.036**
LDL	1.001	0.998–1.004	0.613	LDL	1.001	0.998–1.003	0.614
CHA_2_DS_2_-VASc score	1.313	1.134–1.521	**<0.001**	CHA_2_DS_2_-VASc score	1.230	1.084–1.397	**0.001**
HDL	1.006	0.996–1.017	0.225	HDL	0.998	0.988–1.007	0.601
SYNTAX score	1.009	0.998–1.020	0.111	Clinical SYNTAX score	1.003	1.000–1.007	**0.047**
EF < 60%	1.057	0.794–1.408	0.703	EF < 60%	0.988	0.773–1.264	0.925
GRACE score > 140	1.226	0.942–1.594	0.129	GRACE score > 140	1.024	0.819–1.282	0.832
CrCl	0.987	0.981–0.992	**<0.001**	CrCl	0.994	0.990–0.999	**0.010**
Anemia	1.248	0.974–1.601	0.080	Anemia	1.190	0.958–1.480	0.116
NOAF at 12-month follow-up	1.691	0.959–2.982	0.069	NOAF at 12-month follow-up	2.104	1.296–3.417	**0.003**

Abbreviations: LVDD: left ventricular diastolic dysfunction; HT: hypertension; MI: myocardial infarction; LDL: low-density lipoprotein; HDL: high-density lipoprotein; CHA_2_DS_2_-VASc: congestive heart failure, hypertension, age, diabetes, stroke, vascular disease, sex category score; SYNTAX Score: Synergy Between PCI with Taxus and Cardiac Surgery Score; EF: ejection fraction; GRACE: Global Registry of Acute Coronary Events; CrCl: creatinine clearance (Cockcroft–Gault); NOAF: new-onset atrial fibrillation. Models otherwise match the covariate set of Table 4 Model 1 (mortality) and Table 6 Model 1 (MACEs), with age removed and CHA_2_DS_2_-VASc score added. Bold values indicate statistical significance (*p* < 0.05).

## Data Availability

The data presented in this study are available from the corresponding author upon reasonable request. The data are not publicly available due to ethical and patient privacy restrictions, as the dataset contains patient-level clinical information. The relevant de-identified data can also be provided to the Editorial Office upon request for internal evaluation.

## Abbreviations

The following abbreviations are used in this manuscript:

ACEF	Age, Creatinine, Ejection Fraction
ACS	Acute Coronary Syndrome
AF	Atrial Fibrillation
AMI	Acute Myocardial Infarction
ASA	Acetylsalicylic Acid (Aspirin)
AUC	Area Under the Curve
CABG	Coronary Artery Bypass Grafting
CHA2DS2-VASc	Congestive Heart Failure, Hypertension, Age ≥ 75, Diabetes, Stroke/TIA/Thromboembolism, Vascular Disease, Age 65–74, Sex Category (Female)
CI	Confidence Interval
CICU	Coronary Intensive Care Unit
COVID-19	Coronavirus Disease 2019
CSS	Clinical SYNTAX score
DAPT	Dual Antiplatelet Therapy
DM	Diabetes Mellitus
ECG	Electrocardiography
EF	Ejection Fraction
GRACE	Global Registry of Acute Coronary Events
HDL	High-Density Lipoprotein
CrCl	Creatinine Clearance (Cockcroft–Gault)
HbA1c	Hemoglobin A1c
HR	Hazard Ratio
HT	Hypertension
IQR	Interquartile Range
LA	Left Atrium
LDL	Low-Density Lipoprotein
LVDD	Left Ventricular Diastolic Dysfunction
MACE	Major Adverse Cardiovascular Event
MI	Myocardial Infarction
NSTEMI	Non-ST-Elevation Myocardial Infarction
NOAF	New-Onset Atrial Fibrillation
NOAC	Non-Vitamin K Oral Anticoagulant
OAC	Oral Anticoagulation
OACIS	Osaka Acute Coronary Insufficiency Study
OR	Odds Ratio
PCI	Percutaneous Coronary Intervention
RAAS	Renin-Angiotensin-Aldosterone System
ROC	Receiver Operating Characteristic
SD	Standard Deviation
SIRTAX	Sirolimus-Eluting Stent Compared with Paclitaxel-Eluting Stent for Coronary Revascularization
SS	SYNTAX Score
STEMI	ST-Elevation Myocardial Infarction
SYNTAX	Synergy Between PCI With Taxus and Cardiac Surgery
TAT	Triple Antithrombotic Therapy
TIA	Transient Ischemic Attack
VIF	Variance Inflation Factor
